# Effect of eye care clinical guidelines training on nurses’ knowledge, attitude, and practice and eye complications among critically ill patients: pre and post-study design

**DOI:** 10.1186/s12912-025-03390-5

**Published:** 2025-06-30

**Authors:** Amina Hemida Salem Ghattas

**Affiliations:** Faculty of Nursing, Critical Care & Emergency Nursing Department, Alexandria University, Alexandria, Egypt

**Keywords:** Eye care clinical guidelines, Nurses’ competencies, Patients’ outcomes, Critically ill patients

## Abstract

**Background:**

Eye care plays a vital role in delivering holistic nursing to patients in intensive care units (ICUs), particularly those with impaired ocular defense mechanisms due to factors such as infections, medication use, or mechanical ventilation. Despite its importance, eye care is often overlooked as clinical attention is typically focused on sustaining critical bodily functions. To prevent ocular complications, nurses must be well-educated and proficient in eye assessment, accurate diagnosis, and the application of current evidence-based protocols—skills that can be developed through formal education and ongoing practical training.

**Aim:**

This study aimed to evaluate the impact of implementing an Eye Care Clinical Guidelines (ECCG) training program on nurses’ knowledge, attitudes, and practices, as well as its effect on reducing eye complications in critically ill ICU patients.

**Methods:**

A pretest-posttest quasi-experimental design was used, involving two participant groups: 75 nurses and 100 patients. Key outcomes—nurses’ knowledge, attitudes, and practices, along with the incidence of eye complications—were measured before and after the ECCG training intervention. Comparisons were made between pre- and post-intervention results to assess the effectiveness of the training.

**Results:**

The study included 75 ICU nurses and 100 critically ill patients. Post-intervention, nurses demonstrated significantly improved knowledge and practice scores related to eye care (*P* ≤ 0.001), although changes in attitude scores were not statistically significant (*p* = 0.147). Furthermore, the incidence of eye complications decreased from 84% in the pre-intervention group to 46% in the post-intervention group, showing a highly significant difference (*p* < 0.001).

**Conclusions:**

The ECCG training significantly enhanced ICU nurses’ knowledge and practices and contributed to a marked reduction in eye complications among critically ill patients. It is therefore recommended that Egyptian ICUs adopt standardized eye care guidelines and protocols to improve patient outcomes and the overall quality of care.

**Clinical trial number:**

Not applicable.

## Background

Patients admitted to the intensive care unit are suffering from life-threatening conditions that require comprehensive, collaborative care from a skilled interdisciplinary team. This team plays a crucial role in safeguarding patient outcomes by applying evidence-based assessment and intervention protocols. These protocols significantly improve both physiological and psychological health by minimizing complications, errors, morbidity, mortality, and the overall cost and duration of ICU stays. During the ICU admission, healthcare professionals primarily focus on stabilizing vital functions and managing critical conditions. As a result, less urgent concerns—such as eye complications, particularly ocular surface disorders (OSDs)—are often overlooked, especially by nursing staff [1–5].

Eye care plays a vital role in nursing practice for ICU patients, helping to prevent ocular complications and significantly improving patients’ quality of life after discharge. To ensure effective eye care, nurses must conduct thorough clinical assessments upon admission and at regular intervals throughout the ICU stay. Incorporating routine eye care into standard care bundles has proven effective in reducing eye complications among critically ill patients. Nurses who receive targeted clinical training and consistently follow eye care guidelines contribute to lower rates of ocular issues. Key protective measures include both eye assessments and appropriate interventions. Nurses should evaluate eyelid closure as soon as the patient is admitted, immediately after stabilizing vital functions (airway, breathing, and circulation), and continue these checks regularly. Inadequate eyelid closure, or lagophthalmos, strongly correlates with the onset of eye complications, which can progress to dry eye and corneal ulcers if left unaddressed [5, 6–11].

In Egyptian Intensive Care Units (ICUs), several barriers hinder the effective implementation of eye care protocols. *One of these barriers includes the Knowledge-practice gap.* Several studies have been conducted addressing the gap in the Egyptian ICUs’ nurses’ eye care knowledge and practice. In one study conducted at Talkha Central Hospital and Mansoura University Emergency Hospital revealed that while ICU nurses possessed satisfactory knowledge about eye care, their practical application was inadequate. Essential practices such as handwashing before eye care, assessing signs of infection, and evaluating pupil reflex and eye movement were often neglected. This discrepancy suggests that knowledge alone does not translate into practice, possibly due to factors like high patient-to-nurse ratios and time constraints [12]. In another study conducted at Beni-Suef and Helwan universities revealed that about 74% of the studied nurses had an unsatisfactory level of knowledge regarding eye care, while about 92% of the studied nurses had a negative attitude regarding eye care, and about 6% of the studied nurses had a satisfactory level of practice regarding eye care [7]. 

Another barrier that hinders the effective implementation of eye care protocols in Egyptian ICUs is a *lack of standardized protocols*: A study conducted at Benha University Hospital found that only 10% of nurses had a good level of knowledge, and 20% had satisfactory practices regarding eye care for comatose patients before implementing specific guidelines. Post-implementation, there was a significant improvement, indicating that the absence of standardized protocols contributes to inconsistent practices among nurses [13]. Moreover, another barrier to the implementation of eye care protocols is *Resource Constraints and Training Deficiencies*: The Egyptian healthcare system faces underfunding, with only 4.75% of the Gross Domestic Product (GDP) dedicated to healthcare services. This underinvestment leads to shortages of medical equipment and personnel, affecting the quality of care, including eye care in ICUs. Additionally, limited access to continuous professional development and training programs results in a lack of awareness or understanding of evidence-based eye care protocols among nurses [14]. 

Additionally, there may be *limited access to continuous professional development and training programs as a result of heavy workload (high nurse-to-patient ratios)*, resulting in a lack of awareness or understanding of evidence-based eye care protocols. In some cases, the absence of clear institutional guidelines or standardized policies on eye care further contributes to inconsistent practices. A study involving 207 ICU nurses across hospitals in Fayoum and Damanhour found a significant positive correlation between high workloads and the incidence of missed nursing care. Nurses reported that increased workloads, coupled with inadequate teamwork, led to essential care activities being omitted or delayed, thereby compromising patient safety [15].

Finally, *the lack of performance monitoring and documentation* related to eye care makes it difficult to track adherence and improve practices. Collectively, these barriers highlight the need for systemic changes, including the development of clear protocols, enhanced training, and better resource allocation, to ensure effective eye care in Egyptian ICUs.

### Significance of study

Life-sustaining measures are the top priority in the ICU. Conversely, eye care is a minor consideration for ICU patients. The World Health Organization (2019) reports that at least 2.2 billion people worldwide have near or distance vision impairments, with at least 1 billion of these having preventable or treatable vision impairment. Furthermore, the inclusion of eye care is recommended to be included in national health strategic planning. Nurses, as key health care team members, are pivotal to the provision of holistic care, including the eyes. Thus, nurses need to have considerable competence (knowledge, skills, and attitudes) in carefully assessing and correctly recognizing the patients’ eye problems, and this requires a standardized and evidence-based eye care protocol to be available in the ICU [16, 17].

## Methods

### Aim

Determine the effect of implementing eye care clinical guidelines (ECCG) training on nurses’ knowledge, attitude, and practice, and the reduction of eye complications among the critically ill patients admitted to the ICU.

### Study hypotheses

**H1** Nurses’ knowledge regarding eye care would be improved after attending eye care clinical guidelines training than before.

**H2** Nurses’ practice regarding eye care would be improved after attending eye care clinical guidelines training than before.

**H3** Nurses’ attitudes regarding eye care would be improved after attending eye care clinical guidelines training than before.

**H4** Patients who were cared for by nurses who attended the ECCG exhibit a lower incidence rate of eye complications than patients who were cared for by nurses who did not.

#### Research design

A quasi-experimental design was used to conduct the current study.

**A pretest-posttest quasi-experimental research design** was used to conduct the study on two groups of participants: nurses and patients. As regards the first group of participants, which includes nurses, the same dependent variables (nurses’ knowledge, attitudes, and practice) were assessed or measured before (pretest) and after (posttest) implementation of ECCG training (intervention). The nurses’ scores were measured before and again following the intervention, and the difference between the pre- and post-scores was compared. As regards the second group of participants, patients, the dependent variable (eye complications) was assessed or measured before and after the intervention (implementation of ECCG), and the difference between the pre- and post-intervention incidence of eye complications was compared.

### Sample

- A Convenient sample of all available nurses (75) who worked in the selected setting and had inclusion criteria of working in the ICU for at least 6 months, providing direct patient care, and the nurse was excluded if he/she was absent from more than one of the class sessions, and incomplete questionnaire.
- A purposive sample of adult (≥ 18 years) critically ill patients who were free from ocular surface disorders (dry eye, eye edema, and conjunctival injection) on the first day of the study, had incomplete eyelid closure (grade 1 or 2), and had no significant eye trauma. A total of one hundred patients were included in the study from the previously mentioned setting. Those patients were divided equally into 2 constructed groups: the study group (*n* = 50), who received care after implementing designed eye care guidelines, and the control group (*n* = 50), who received routine hospital care before implementing eye care guidelines. The sample size was estimated using G power (version 3.1.5) for one-way ANOVA, 95% power, a medium effect size of 0.25, and an alpha level of ≤ 0.05. According to this formula, the minimum required sample size was eighty-five patients. The population was oversampled to account for the possible attrition rate of the patients. However, one hundred patients were included in the sample.

### Setting

A two-phase study (pre- & post) was conducted in the intensive care units, namely Unit III at the Main University Hospital. This unit consists of two halls and receives critically ill patients who require support for the body’s vital functions. Each hall consists of 12 beds, receiving critically ill patients who require ongoing and intensive assessment and care.

### Tools

The following three tools were used to collect data relevant to the current study:

#### Tool one: eye care clinical competency questionnaire. This tool comprises 4 parts

##### Part I

This part was developed by the researcher to elicit the sociodemographic and clinical data of the nurses, including age, sex, and work experience in the ICU, level of education, available eye care protocol, and attending the specialized eye care course.

##### Part II

The eye care clinical competency questionnaire (knowledge domain) was adopted from Ebadi et al. (2017) [18]. This part was used to assess the nurses’ knowledge. The knowledge domain consists of eighteen 18 multiple-choice questions about the eye, eye care, and iatrogenic eye conditions (anatomy, physiology, risk factors of eye injury in the ICU, common complications of the eye in the ICU, treatment, and nursing practices) in critically ill patients. Each question has 4 distractors with only one correct choice, and the participants were asked to choose the correct answer. The knowledge domain is scored with 1 (correct answer) or 0 (incorrect answer). The possible score range is 0–18. Nurses’ total knowledge was classified into ≥ 75% was considered a good level of knowledge, a score of 60–<75% was considered an average level of knowledge, while those who obtained a score of < 60% were considered a poor level of knowledge.

##### Part III

The eye care clinical competency questionnaire (attitudes domain) was adopted from Ebadi et al. (2015). This part is dedicated to assessing the nurses’ attitudes toward the importance of eye care. It is comprised of 7 items. A five-point Likert scale is used for the attitude’s domain: 1 = not important to 5 = very important. The minimal score was 7, and the maximal score was 35. Scores ≥ 60% indicate a positive attitude, while scores < 60% indicate a negative attitude.

##### Part IV: evidence-based clinical eye care competency observational checklist

The eye clinical care guidelines observational checklist was developed by the researcher after reviewing related literature and converted to an observation checklist form [18–20]. To assess the eye care nurses’ performance of eye care practices. It is comprised of two themes: assessment (5 items) and intervention (8 items). Each nurse was observed while he/she was performing the eye care procedure. The three-point Likert scale was used to assess the level of nurses’ performance: (0) = not done or done incorrectly = [1] = done, correct but incomplete, and [2] = done correctly and complete. The minimal score was 0, and the maximal score was 66. A high score indicates a high level of performance. Also, the degree of performance was categorized into two categories based on the aggregate scores: competent level of practice (≥ 80%) and incompetent level of practice (< 80%). This tool was assessed for its content and face validity by 5 experts in the field of critical care nursing (clinical and academic) and ophthalmology, and it was pre-tested for internal reliability by a group of nurses (*n* = 10) who were not involved in the study. The reliability of the tools was assessed by Cronbach’s alpha (α = 0.81) for the entire questionnaire, for the knowledge domain (0.80), attitudes domain (0.76), and nursing practice domain (0.86).

#### Tool two: eyelid closure assessment sheet

This tool consists of two parts.

##### Part I

This part was developed by the researcher to elicit the sociodemographic and clinical data of the patient, including age, sex, Glasgow Coma Scale (GCS), Diagnosis, positive pressure ventilation, sedation, central venous pressure, and APACHE II.

##### Part II: grading eyelid closure (lagophthalmos scale)

This part was developed and was clinically used to classify the degree of lagophthalmos which ranges from 0 to 2 as follows; grade 0 means that lids are completely closed, grade 1 reflects any conjunctival exposure (any white of the eye being visible) but no corneal exposure and grade 2 that relate to any corneal exposure, even a very tiny amount. The Kappa score indicated significant reliability of the Lagophthalmos Scale (0.88).

#### Tool three: superficial ocular surface disorder assessment scales package

This tool consists of three scales that assess the occurrence of eye problems:

##### Scale I: surgeon periorbital rating of edema (SPRE)

This tool was developed and validated by a group of surgeons to assess the levels of eye edema [21]. The scale consists of four grades to assess the severity of periorbital edema. Grade 1 regards no coverage of the iris with eyelids; grade 2 means that there is slight coverage of the iris with swollen eyelids. Grade 3 indicates full coverage of the iris with swollen eyelids, and grade 4 reflects full closure of the eye.

##### Scale II: eye dryness schirmer

Eye dryness. The Schirmer scale is a scale for determining the degree of eye dryness based on the result of the Schirmer test, which involves placing a strip that is put in the patient’s eye and then becomes wet. The centimeters of wetting of the strip determine the severity of eye dryness. The scale includes three categories. The least one ranges from 0 to 5, which assumes severe dry eye. The second category ranges from 5 to 10 of wetting of the strip, which indicates moderate dryness of the eye. The highest one ranges from 10 to 15 mm, which reflects mild dryness of the eye. The Kappa score indicated significant reliability (0.80) for the eye dryness Schirmer scale.

##### Scale III: conjunctival injection

The conjunctival injection assessment scale consists of four grades, ranging from 0 to 3, that classify the grade of the conjunctival injection. Grade 0 refers to the fact that there is no redness of the eye, and grade 1 indicates some redness. Grade 2 means extensive redness of the eye, and grade 3 reflects overall conjunctival redness. The Kappa score indicated significant reliability (0.89) for the Conjunctival Injection scale.

## Methodology

### Phase I: pre-implementation of eye care guidelines educational sessions

- Each nurse was interviewed individually by the researcher to explain the aim of the plan of the study and to collect the baseline sociodemographic and clinical data of the nurses. Written informed consent was also obtained.
- Each nurse was asked to complete the knowledge and attitude domains of the EC clinical competency questionnaire (Tool I; Parts I, II, & III). The average time needed for the completion of each interview for nurses was between 30 and 45 min. The questionnaire was handed in and collected by the researcher.
- Nurses’ performance of routine care was observed by the researcher daily for 7 consecutive days (tool one: part IV). No follow-up was performed after the patient’s discharge from the ICU.
- The patient’s sociodemographic and clinical data were collected by the researcher. (tool two; part I).
- The time zero of the patient’s assessment was the day of admission to the ICU (initial assessment, eye assessment for the presence of risk factors, especially Lagophthalmos (tool two part II)), and the follow-up assessment time was seven days. This follow-up time was chosen because superficial eye disorders occurred around the second to the seventh day of the patient’s admission.

### Phase II: eye care guidelines educational sessions

- Before the educational sessions, necessary arrangements for educational classes, such as place, time, educational package, and catering, were made.
- Content validity of the educational content was done by a panel of nursing educators and ophthalmologists. Suggested modifications were made accordingly.
- An ophthalmologist and infection control specialist were invited to present the specialized information.
- The duration of theory sessions was 45 to 60 min for each session, and practical sessions ranged between 45 and 60 min for three days/week.
- At the beginning of each session, the researcher started with a summary of what was covered in the previous session and the objectives of the new one, taking into consideration using simple and clear language to suit the nurses. Different teaching methods were used, including lectures, discussions, flip charts, brainstorming, demonstration, and re-demonstration. The teaching aids used were videos and PowerPoint presentations (Table 1).

### **Phase III: post-implementation of eye care educational sessions**

- After the end of the training sessions, the same ECCG questionnaire was re-evaluated. The nurses’ eye care practice items were assessed by the researcher in three different work shifts for each nurse by using the same pretest observational checklist.
- For seven consecutive days, an assessment of each patient was carried out once daily in the morning shift by the researcher using a portable slit lamp to evaluate the effect of eye care practice provided by the nurses on the occurrence of ocular surface disorders (dry eye, periorbital edema, and conjunctival injection) (tool three). This action was done to evaluate the effect of eye care provided by the nurses.
- The nurses’ eye care practice items were assessed by the researcher in three different work shifts for each nurse.

**Table 1:** Schedule of eye care educational training sessions

Session #	Instructor	Educational Content
Day 1 Session (1)	Researcher	− Welcome the participants. − Explain the aims and objectives of the session. − Clarify why critically ill patients are at great risk of developing eye complications.
	Ophthalmologist	− Describe the anatomical structure of the eye and the functions of each structure. − Explain the physiological and mechanical protective mechanisms that protect the eye from injury and infection. − Explain the causes and pathophysiology that could impair each defense mechanism using colorful, illustrative pictures and videos.
Day 2 Session (1)	Ophthalmologist	− Explain the common eye disorders and the pathophysiology of each.
		− Explain the eye examinations and medications used in different eye disorders.
Day 2 Session (1)	Infection Control Specialist	− Review the infection control steps while performing eye hygiene.
Day 2 Session (2)	Ophthalmologist	− Demonstrate how to use the different tools used in eye assessment (grading of Lagophthalmos, Surgeon Periorbital Rating of Edema Scale (SPRES, Eye Dryness Schirmer & Conjunctival Injection Scale).
Day 3 Session (1)	Researcher	− Explain and review in detail the assessment of the eye. − Review of the latest guidelines and research on eye disorders in the intensive care unit. − Review eye hygiene, methods for closure of the eyelids, eye assessment methods, eyelid position, and EC in practice.
Day 3 Session (2)	Researcher	− Demonstrate a hands-on eye care procedure. − Each nurse redemonstrates the eye care procedure on a real eye.

### Ethical considerations

Ethical approval to conduct the proposed study was obtained from the Ethical Research Committee at the Faculty of Nursing, Alexandria University, and the approval to collect the data was obtained from the Hospital and CCU authorities. Additionally, informed consent was obtained from the participants who agreed to participate and meet the inclusion criteria. Voluntary participation and withdrawal from the study at any time without any rationale or penalty, and confidentiality of the information were assured. The anonymity of the participants was maintained.

### Data analysis

The collected data were categorized, analyzed, and tabulated using the SPSS computer program Version 21. Numerical data were represented as mean and standard deviation. Qualitative data were represented as frequency and percentage. A parametric Chi-square test was used to compare qualitative variables. The correlation between variables was assessed using the Pearson correlation coefficient. A difference was deemed statistically significant at a p-value < 0.05, highly statistically significant at a p-value ≤ 0.001, and not statistically significant at a p-value > 0.05.

## Results

After compiling all the phases of the study, the data were statistically analyzed, and the following results were presented as the following:

Table 2 shows the sociodemographic and clinical data of nurses who participated in the study. It was noted that the mean age of the studied nurses was 29.95 ± 4.93years, and the majority (70.7%) of them were females. As regards nurses’ years of experience, nurses who had more than 6 months and less than 5 years were represented equally with nurses who had more than 10 years (29.3%). While the percentage of nurses who had years of experience between 6 and 10 years was 41.4%. About academic qualifications, 60% of them had a diploma degree and 40% had a bachelor’s degree. Additionally, more than half of the sample (73.3%) reported that no eye protocol is available at the unit; however, 26.7% was not known. Moreover, the majority of the nurses (84%) did not attend any previous training courses about eye care for critically ill patients.

**Table 2:** Sociodemographic and clinical data of nurses who participated in the study

Variable	Frequency	Percent
Age		
≥ 18–25	34	45.3
26–35	25	33.3
36 ≥ 45	16	21.4
M ± SD	29.95 ± 4.93	
Sex		
Male	22	29.3
Female	53	70.7
Years of Experience		
> 6 months - ≤ 5 years	22	29.3
6 years – 10 years	31	41.4
> 10 years	22	29.3
Level of Education		
Diploma	45	60
Bachelor	30	40
Is there an eye care protocol in the unit?		
Yes	0	0
No	55	73.3
I do not know	20	26.7
Did you attend any eye care educational activities?		
Yes	12	16
No	63	84

Table 3 reveals a comparison between the percentage score of the nurses’ Knowledge before and after attending ECCG. It was observed that about two-thirds (65.3%) of the studied nurses had poor knowledge before the implementation of ECCG, compared to 5.4% after the implementation of ECCG. The difference between pre and post-ECCG nurses’ knowledge was statistically significant (*P* < 0.05).

**Table 3:** Comparison of percent score of the nurses’ knowledge before and after the implementation of ECCG

Level of Knowledge	Pre (75)		Post (75)		χ^2^	P -value
	No	%	No	%		
Good (≥ 75%)	9	12	52	69.3	68.630	0.000**
Average (60% - < 75%)	17	22.7	19	25.3		
Poor (< 60%)	49	65.3	4	5.4		

Table 4 shows a comparison between the percentage score of the nurses’ attitudes before and after attending ECCG. It was noted that the majority of the sample had good attitudes toward eye care (88%) before the implementation of ECCG, compared to 94.7% after the implementation of ECCG. The difference between pre and post-ECCG nurses’ attitudes was not statistically significant (*P* < 0.05).

**Table 4:** Comparison of mean scores of the nurses’ attitudes before and after the implementation of ECCG

Level of Attitudes	Pre (75)		Post (75)		χ^2^	P -value
	No	%	No	%		
Positive ≥ 60%	66	88	71	94.7	2.1065	0.147
Poor ≤ 60%	9	12	4	5.3		

**A highly statistically significant P value, *P* < 0.001

Table 5 shows a comparison between the mean score of the nurses’ attitudes before and after attending ECCG. It was found that the vast majority of the nurses had competent eye care performance post-implementation of ECCG (84.0%) compared to pre-implementation of ECCG (18.7%) pre-implementation of ECCG. The difference between the pre and post-ECCG was highly statistically significant at p-value (*P* < 0.001).

**Table 5:** Comparison of percent scores of participants’ level of performance before and after the implementation of clinical training guidelines

Level of Performance	Pre (75)		Post (75)		χ^2^	P -value
	No	%	No	%		
Competent Practice (≥ 85)	14	18.7	63	84	49.759	0.000**
Incompetent Practice (< 85	61	81.3	12	16		

**A highly statistically significant P value, *P* < 0.001

Table 6 displays the distribution of sociodemographic and clinical data of the patients included in the study, Pre & Post ECCG. Regarding gender, it can be noted that more than half of the patients in the pre and post-ECCG groups were male, with 58% and 52%, respectively. Regarding the patient’s age, the mean age for the pre-ECCG group was 57.82 ± 16.57compared with 61.12 ± 17.65 for the post-ECCG group. In conclusion, there were no statistically significant differences between the two groups regarding all patients’ sociodemographic data. As regards the clinical data, it was observed that the majority of patients in the pre and post-groups (80%, and 78% respectively) were attached to mechanical ventilation on synchronized Intermittent Mandatory Ventilation (SIMV) mode and 62% of the patients in the pre-group compared with 48% for the post group were on sedation without neuromuscular blockers. Regarding APACHI II, the mean score was higher in the post-group (28.54 ± 4.95) than the pre-group (27.22 ± 4.85), with no statistically significant difference between the two studied groups. Regarding GCS, the mean score was 4.61 ± 1.35 in the pre-group compared with 4.35 ± 1.22 in the post-group.

**Table 6:** Distribution of sociodemographic, clinical data of the patients included in the study pre & post ECCG

Variables	Pre (*n* = 50)		Post (*n* = 50)		Test of sig.	*p*
**Age in years**						
Mean ± SD	57.82 ± 16.57		61.12 ± 17.65		t = 0.964	0.337
**Sex**	No.	%	No.	%		
Male	29	58.0%	26	52.0%	χ^2^ = 0.364	0.546
Female	21	42.0%	24	48.0%		
**GCS**						
Mean ± SD	4.61 ± 1.35		4.35 ± 1.22		t = 1.026	0.307
**Sedation**	No.	%	No.	%		
Patients with sedation only	31	62.0%	24	48.0%	χ^2^ = 1.980	0.159
Patient with sedation and neuromuscular blockers	19	38.0%	26	52.0%		
**APACHE score**						
Mean ± SD	27.22 ± 4.85		28.54 ± 4.95		t = 1.346	0.181
**CVP**						
Mean ± SD	13.13 ± 4.86		14.77 ± 6.59		t = 1.419	0.159
**Attached to MV**						
SIMV	40	80.0%	39	78.0%	χ^2^ = 3.898	^MC^*p* = 0.273
AC	2	4.0%	0	0.0%		
CMV	1	2.0%	3	6.0%		
PCAC	7	14.0%	8	16.0%		
**PEEP**						
Mean ± SD	5.19 ± 0.38		5.36 ± 0.76		t = 1.475	0.145
**Peak pressure**						
Mean ± SD	66.14 ± 11.08		67.63 ± 6.86		t = 0.807	0.422

Table 7 shows how the control and study groups are distributed based on the severity of lagophthalmos. It indicates that the majority of patients in both the control and study groups experienced grade [2] lagophthalmos (corneal exposure), with percentages of 58% and 70%, respectively, revealing no significant difference (*p* < 0.221) between the two groups examined.

**Table 7:** Distribution of the control and study groups according to the degree of lagophthalmos

Degree of lagophthalmos	Control (*n* = 50)		Study (*n* = 50)		χ^2^	p
	No.	%	No.	%		
− Conjunctival exposure (grade 1) on admission	21	42.0%	15	30.0%	1.563	0.221
− Corneal exposure (grade 2) on admission	29	58.0%	35	70.0%		

χ^2^: Chi-square test *p* < 0.05

Table 8 illustrates the rate of eye dryness among patients in both study groups. The table indicates that the occurrence of severe eye dryness rose from 0% on the first day of the study to 44% on the 4th, 5th, and 6th days, reaching 58% on the 7th day for the control group. Additionally, it can be observed from the same table that in the study group, severe eye dryness increased from 0% on the first day to 18% on both the 6th and 7th days. Furthermore, the current table shows a significant difference (*p* < 0.001) regarding eye dryness between the two groups from the 2nd day through to the 7th day of the study.

**Table 8:** Incidence rate of eye dryness among the control and study groups

Day of the exam	Eye dryness grade	Control (*n* = 50)		Study (*n* = 50)		χ^2^	^MC^ *p*
		No.	%	No.	%		
1st day	Mild	50	100%	50	100%	-	-
	Moderate	0	0.0%	0	0.0%		
	Severe	0	0.0%	0	0.0%		
2nd day	Mild	29	58.0%	50	100%	26.582^*^	< 0.001^*^
	Moderate	21	42.0%	0	0.0%		
	Severe	0	0.0%	0	0.0%		
3rd day	Mild	28	56.0%	50	100%	28.205^*^	< 0.001^*^
	Moderate	22	44.0%	0	0.0%		
	Severe	0	0.0%	0	0.0%		
4th day	Mild	12	24.0%	37	74.0%	35.065^*^	< 0.001^*^
	Moderate	16	32.0%	13	26.0%		
	Severe	22	44.0%	0	0.0%		
5th day	Mild	12	24.0%	37	74.0%	35.065^*^	< 0.001^*^
	Moderate	16	32.0%	13	26.0%		
	Severe	22	44.0%	0	0.0%		
6th day	Mild	12	24.0%	31	62.0%	15.232^*^	< 0.001^*^
	Moderate	16	32.0%	10	20.0%		
	Severe	22	44.0%	9	18.0%		
7th day	Mild	12	24.0%	31	62.0%	18.974^*^	< 0.001^*^
	Moderate	9	18.0%	10	20.0%		
	Severe	29	58.0%	9	18.0%		
Fr		219.475^*^		99.957^*^			
P		< 0.001^*^		< 0.001^*^			

χ^2^: Chi-square test MC: Monte Carlo

Fr: Friedman Test to compare the change between the different days in each group

*: Statistically significant at *p* ≤ 0.05

Table 9 presents the occurrence rate of periorbital edema in both the control and study groups. This table shows that on the first day, neither of the groups exhibited any signs of periorbital edema (0%). By the seventh day, the incidence of Grade 3 periorbital edema rose to 46% for the control group, while it was 20% for the study group, revealing a statistically significant difference between the two (p-value = 0.021). Additionally, the prevalence of periorbital edema was notably greater in the control group compared to the study group throughout the study.

**Table 9:** Incidence rate of periorbital edema among the patients of the control and study groups

Day of the exam	Edema scale	Control (*n* = 50)		Study (*n* = 50)		χ^2^	^MC^ *p*
		No.	%	No.	%		
1st day	Grade 1	44	88.0%	50	100%	6.383^*^	0.027^*^
	Grade 2	6	12.0%	0	0.0%		
	Grade 3	0	0.0%	0	0.0%		
	Grade 4	0	0.0%	0	0.0%		
2nd day	Grade 1	25	50.0%	49	98.0%	32.453^*^	< 0.001^*^
	Grade 2	22	44.0%	1	2.0%		
	Grade 3	3	6.0%	0	0.0%		
	Grade 4	0	0.0%	0	0.0%		
3rd day	Grade 1	20	40.0%	42	84.0%	22.030^*^	< 0.001^*^
	Grade 2	24	48.0%	8	16.0%		
	Grade 3	6	12.0%	0	0.0%		
	Grade 4	0	0.0%	0	0.0%		
4th day	Grade 1	20	40.0%	36	72.0%	13.010^*^	0.001^*^
	Grade 2	13	26.0%	10	20.0%		
	Grade 3	17	34.0%	4	8.0%		
	Grade 4	0	0.0%	0	0.0%		
5th day	Grade 1	20	40.0%	34	68.0%	10.954^*^	0.004^*^
	Grade 2	7	14.0%	8	16.0%		
	Grade 3	23	46.0%	8	16.0%		
	Grade 4	0	0.0%	0	0.0%		
6th day	Grade 1	20	40.0%	31	62.0%	10.520^*^	0.005^*^
	Grade 2	7	14.0%	11	22.0%		
	Grade 3	23	46.0%	8	16.0%		
	Grade 4	0	0.0%	0	0.0%		
7th day	Grade 1	20	40.0%	31	62.0%	7.744^*^	0.021^*^
	Grade 2	7	14.0%	9	18.0%		
	Grade 3	23	46.0%	10	20.0%		
	Grade 4	0	0.0%	0	0.0%		
Fr		133.538^*^		91.507^*^			
P		< 0.001^*^		< 0.001^*^			

χ^2^: Chi-square test MC: Monte Carlo

Fr: Friedman Test to compare the change between the different days in each group

*: Statistically significant at *p* ≤ 0.05

Table 10 illustrates the occurrence rate of conjunctival injection among patients in the two groups that were examined. This table indicates a notable distinction between the two groups concerning the occurrence of conjunctival injection throughout the study. It was noted that from the 3rd to the 7th day of the study, the control group exhibited considerably greater percentages of grade 1 conjunctival injection compared to the study group (32%, 42%, 46%, 48%, and 48.0%) in contrast to (8%, 28%, 26%, 28%, and 28%) respectively.

**Table 10:** Incidence rate of conjunctival injection among the patients of the control and study groups

Day of the exam	Conjunctival Injection Scale	Control (*n* = 50)		Study (*n* = 50)		χ^2^	^MC^ *p*
		No.	%	No.	%		
1st day	Grade 0	49	98.0%	50	100%	1.010	^FE^*p* = 1.000
	Grade 1	1	2.0%	0	0.0%		
	Grade 2	0	0.0%	0	0.0%		
	Grade 3	0	0.0%	0	0.0%		
2nd day	Grade 0	43	86.0%	50	100%	7.531^*^	0.016^*^
	Grade 1	6	12.0%	0	0.0%		
	Grade 2	1	2.0%	0	0.0%		
	Grade 3	0	0.0%	0	0.0%		
3rd day	Grade 0	32	64.0%	46	92.0%	11.430^*^	0.002^*^
	Grade 1	16	32.0%	4	8.0%		
	Grade 2	2	4.0%	0	0.0%		
	Grade 3	0	0.0%	0	0.0%		
4th day	Grade 0	26	52.0%	36	72.0%	5.589^*^	0.038^*^
	Grade 1	21	42.0%	14	28.0%		
	Grade 2	3	6.0%	0	0.0%		
	Grade 3	0	0.0%	0	0.0%		
5th day	Grade 0	22	44.0%	36	72.0%	8.598^*^	0.012^*^
	Grade 1	23	46.0%	13	26.0%		
	Grade 2	5	10.0%	1	2.0%		
	Grade 3	0	0.0%	0	0.0%		
6th day	Grade 0	21	42.0%	35	70.0%	8.574^*^	0.013^*^
	Grade 1	24	48.0%	14	28.0%		
	Grade 2	5	10.0%	1	2.0%		
	Grade 3	0	0.0%	0	0.0%		
7th day	Grade 0	21	42.0%	34	68.0%	6.872^*^	0.035^*^
	Grade 1	24	48.0%	14	28.0%		
	Grade 2	5	10.0%	2	4.0%		
	Grade 3	0	0.0%	0	0.0%		
Fr		125.989^*^		71.302^*^			
P		< 0.001^*^		< 0.001^*^			

χ^2^: Chi-square test MC: Monte Carlo

Fr: Friedman Test to compare the change between the different days in each group

*: Statistically significant at *p* ≤ 0.05

Table 11 displays the rate of conjunctival discharge among patients in the two groups analyzed. This table indicates that 24% of individuals in the control group and 14% in the study group exhibited first-degree eye discharge starting from the first day, with no statistically significant difference between the two groups (*p* = 0.202). Additionally, the table shows that by the seventh day of the study, 72% of patients in the control group had experienced grade 3 eye discharge, in contrast to only 14% of those in the study group. Moreover, this table highlights a significant difference (*p* < 0.001) between the two groups from the second to the seventh day of the study regarding grade 3 eye discharge.

**Table 11:** Incidence rate of conjunctival discharge among the patients of the control and study groups

Day of the exam	Conjunctival Discharge Scale	Control (*n* = 50)		Study (*n* = 50)		χ^2^	^MC^ *p*
		No.	%	No.	%		
1st day	Grade 0	38	76.0%	43	86.0%	1.624	0.202
	Grade 1	12	24.0%	7	14.0%		
	Grade 2	0	0.0%	0	0.0%		
	Grade 3	0	0.0%	0	0.0%		
2nd day	Grade 0	14	28.0%	43	86.0%	42.610^*^	< 0.001^*^
	Grade 1	19	38.0%	0	0.0%		
	Grade 2	15	30.0%	7	14.0%		
	Grade 3	2	4.0%	0	0.0%		
3rd day	Grade 0	10	20.0%	41	82.0%	52.506^*^	< 0.001^*^
	Grade 1	5	10.0%	2	4.0%		
	Grade 2	25	50.0%	0	0.0%		
	Grade 3	10	20.0%	7	14.0%		
4th day	Grade 0	10	20.0%	41	82.0%	39.871^*^	< 0.001^*^
	Grade 1	3	6.0%	0	0.0%		
	Grade 2	8	16.0%	2	4.0%		
	Grade 3	29	58.0%	7	14.0%		
5th day	Grade 0	10	20.0%	35	70.0%	33.246^*^	< 0.001^*^
	Grade 1	1	2.0%	3	6.0%		
	Grade 2	6	12.0%	5	10.0%		
	Grade 3	33	66.0%	7	14.0%		
6th day	Grade 0	10	20.0%	33	66.0%	36.465^*^	< 0.001^*^
	Grade 1	1	2.0%	5	10.0%		
	Grade 2	3	6.0%	5	10.0%		
	Grade 3	36	72.0%	7	14.0%		
7th day	Grade 0	10	20.0%	29	58.0%	36.626^*^	< 0.001^*^
	Grade 1	1	2.0%	8	16.0%		
	Grade 2	3	6.0%	6	12.0%		
	Grade 3	36	72.0%	7	14.0%		
Fr		206.107^*^		81.749^*^			
P		< 0.001^*^		< 0.001^*^			

χ^2^: Chi-square test MC: Monte Carlo

Fr: Friedman Test to compare the change between the different days in each group

*: Statistically significant at *p* ≤ 0.05

**Fig. 1:**
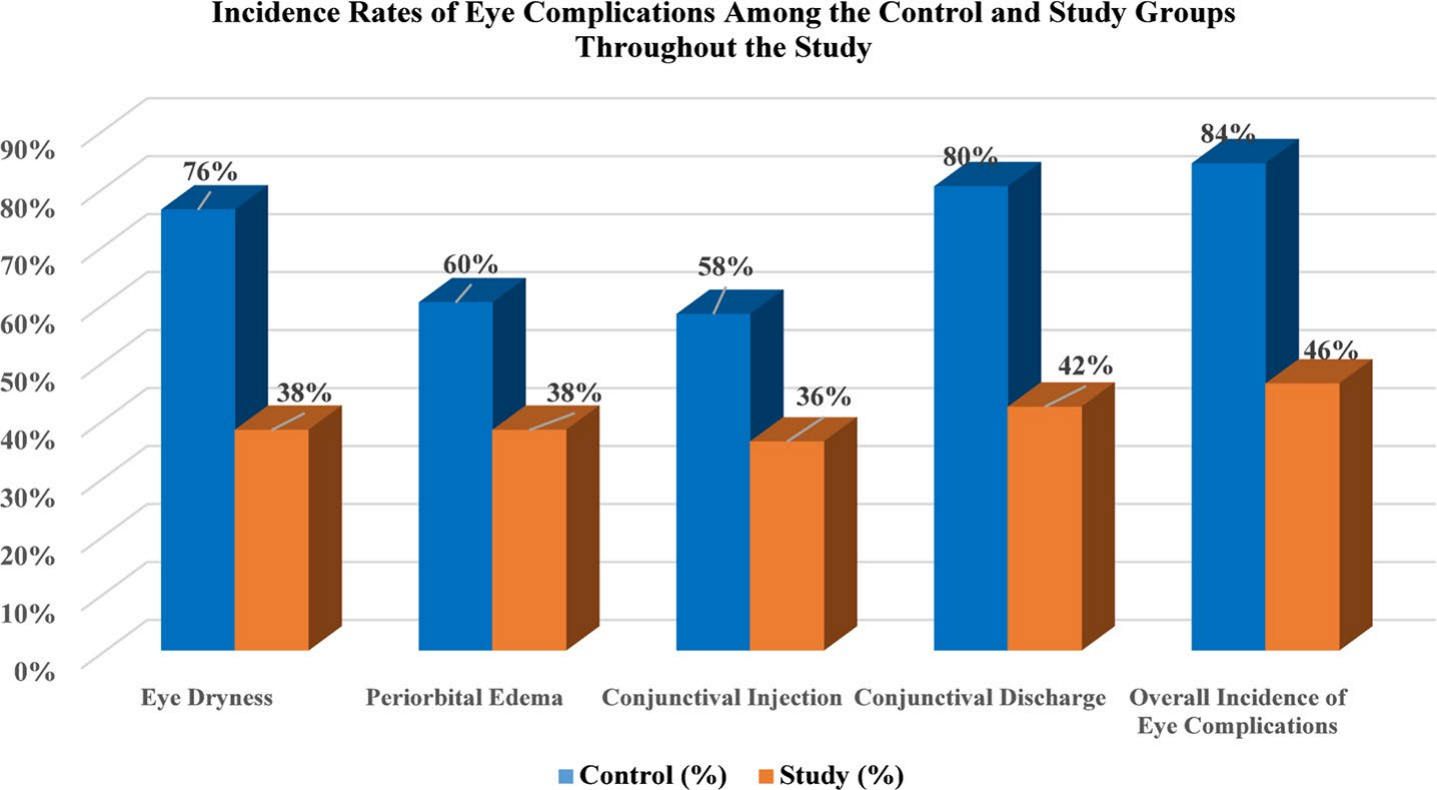
Comparison of the incidence rates of eye complications among both groups throughout the study

Fig. 1 illustrates the incidence rates of eye complications (EC) in the two investigated groups throughout the study. The highest occurrence of EC was observed in eye discharge, affecting 80% of individuals in the control group compared to 42% in the study group. Conversely, the lowest occurrence was noted for conjunctival injection, affecting 58% of participants in the control group and 36% in the study group. Furthermore, the overall incidence of all eye complications was recorded at 84% among patients in the control group and 46% among those in the study group, revealing a highly significant difference (*p* < 0.001) between the two groups examined.

## Discussion

Vision is among the most essential senses for humans. Improper eye care (EC) for patients who have compromised eye protective mechanisms can result in significant ocular problems and potential loss of vision. Insufficient knowledge, attitude, and skills of nurses are considered a barrier to providing EC in the ICU. The present study aimed to determine the effect of implementing eye care clinical guidelines (ECCG) training on nurses’ knowledge, attitude, and practice, and the reduction of eye complications among the critically ill patients admitted to the ICU. To fulfill the aim of the current study, 75 nurses who worked in the ICUs and 100 critically ill patients were recruited.

### Research hypotheses

The present study hypothesized that nurses’ knowledge, practice, and attitude regarding eye care would be improved after attending eye care clinical guidelines training than before. Additionally, the study hypothesized that patients who were cared for by nurses who attended the ECCG exhibit a lower incidence rate of eye complications than patients who were cared for by nurses who did not. To refute these hypotheses, a total of 75 nurses who worked the selected units, and 100 clinically eligible critically ill patients were recruited. Findings revealed that nearly half of the study sample was aged 18–25 years, with a mean age of 29.95 ± 4.93. Additionally, the majority of the nurses were females with a diploma degree. Moreover, the majority of the nurses reported that there was no standardized eye care protocol to be followed in the unit, while the other nurses did not know if there was an available eye care protocol or not. Also, the majority of nurses did not attend any specialized eye care training or instructions. The following section of the discussion will compare the nurses’ knowledge, practice, attitudes, and incidence of eye complications before the implementation of the eye care clinical training guideline, and the same variables (nurses’ knowledge, skills, attitudes, and incidence of eye complications) after the implementation of the eye care clinical training guideline.

### Pre-implementation of ECCG

As regards the percentage scores of pre-implementation ECCG nurses’ eye care knowledge and practice, the finding of the current study reveals that the nurses exhibited low levels of eye care related knowledge (12% had good knowledge, 22.7% had average, and 65.3% of the nurses had poor knowledge) and skills (81.3% had incompetent eye care and 18.7% had competent eye care practice). Moreover, the low levels of eye care nurses’ knowledge and skills are evident in the occurrence of eye complications among the critically ill patients admitted to the ICU (84% of patients have developed eye complications). Low levels of nurses’ knowledge and incompetent practice could be attributed to a lack of in-service education and training, a lack of awareness about implementing eye care, and the eye protocols or guidelines about eye care were not available in the ICU. In addition to the inadequately trained eye care nurses, nurses did not know how to perform eye assessment, the risk factors, and preventive care for ocular complications caused by patients’ admission to the ICU. In addition, nurses pay great attention to managing other life-threatening conditions and maintaining the functions of vital organs, so the subsequent little attention is given to eye care, and eye care is often seen as a relatively minor concern. Other factors, such as high workload and the shortage of nurses, result in unsafe and unprincipled care in the ICU. The high percentage of eye complications could be explained by, in addition to low levels of nurses’ eye care knowledge and skills, the factors that make these patients more vulnerable to the development of eye complications, such as loss of normal protective mechanisms of the eyes, such as decreased tear production and eye blinking reflexes.

The findings of the current study are congruent with several other studies that describe the level of nurses’ knowledge using different adjectives such as poor, bad, and unsatisfactory. A study by Lami & Ayed confirmed that the nurses had poor knowledge and inadequate practice of eye care for patients in the ICU [9]. In the same line, Thomas et al. found that 78% had average knowledge, 50% showed a good attitude, with an average practice pattern was shown by 54% [22] Additionally, Ebadi et al. concluded that nurses’ knowledge in eye care for ICU patients was moderate, and their attitude and practice were good. Accordingly, developing and implementing continuing education programs to promote their eye care knowledge, attitude, and practice is strongly recommended [23]. In another study conducted by Flatela et al. mentioned that nurses showed poor knowledge and practices of common eye conditions. There is an urgent need for eye health training and clear management protocols on eye care [24]. In addition to a study that was conducted by Hashim, who revealed that the majority of the respondents had an unsatisfactory level of knowledge (94.1%). In contrast, the majority of the respondents had a satisfactory level of attitude (94.1%) [25]. Moreover, Sayed et al. reported that only about 10% of the studied nurses had a satisfactory level of practice regarding eye care [6]. Similarly, Jaafr et al. found that the total mean score of nurses’ knowledge was poor (0.33) [26]. Contrary to the results of the current study, Mehrjardi, Mohamed & El-dakhakhny, Khalil, and Vyas in their studies found that the nurses had good, high, and sufficient eye care-related knowledge [13, 27–29]. 

Despite the low levels of the nurses’ knowledge and practice in eye care, a promising finding of the study was that nurses had a positive attitude toward the importance of eye care despite having limited eye care knowledge and skills. The contradiction between the poor nurses’ knowledge, incompetent practice, and high or positive nurses’ attitudes toward eye care could be explained by a self-report questionnaire that was used to assess their attitudes (how the nurses perceived the importance of eye care in critically ill patients). This type of questionnaire allows the nurses to subjectively select their responses, which may be either overestimated or underestimated. Another reason behind the positive nurses’ attitudes may be nurses’ worries about their annual evaluation.

### Post-implementation of ECCG

The implementation of ECCG has significantly improved nurses’ knowledge, practice, and attitudes. This is demonstrated by the significant difference between the percentage of nurses’ knowledge and practice scores. After attending ECCG, the nurses who had a good level of knowledge before attending ECCG statistically improved (12% versus 69.3%). Similarly, there was a statistically significant improvement in the percentage score of the levels of nurses’ practice post-ECCG (14.6% versus 84%). However, the improvement in the mean score of nurses’ attitudes was not statistically significant (88% versus 94.7%). Another piece of evidence that supports the fact of improvement in nurses’ competencies is the reduction in the incidence of eye complications among the studied patients.

The findings are directly in line with the findings of studies conducted by Dawood & Bakey, who assessed the nurses’ clinical competency in providing eye care for patients with altered consciousness in intensive care units. They found low and moderate level mean at the pre-test to high level of knowledge mean at posttest time duration, shifting in the mean score to very high attitudes at the post-test time, significant shifting in the mean of score of practice from low level to high level which revealed improving in nurses’ practices about eye care [30]. Moreover, Gungor et al. aimed to determine ICU nurses’ knowledge, attitudes, and practices regarding eye care. The clinical competency levels of ICU nurses in eye care improve with post-graduation education, the use of protocols, and updated information [31]. Mehrjardi et al. concluded that training nurses based on EC clinical guidelines for anesthetized patients can improve the knowledge, attitude, and practice of ICU nurses [32]. Similarly, Tork et al. found that nurses’ knowledge and practice had significantly improved following the implementation of designed eye health protocols [33]. A similar conclusion was reached by Liem, Elkasby et al., and Mehrjardi et al. [34, 35, 36].

Concerning the effect of ECCG on the incidence of eye complications, the current study demonstrated that the overall incidence of eye complications was reduced significantly from 84 to 46%. A similar conclusion was reached by Mobarez et al., they have showed a reduction in keratitis, conjunctivitis, dry eye, and corneal ulceration when patients are treated with an Eye Care Protocol. Furthermore, Pourghaffari Lahiji et al. have reported that it has been demonstrated that the implementation of a protocol for eye care significantly reduced ocular complications. Similarly, Sama et al. concluded that applying a protocolized eyecare bundle has significantly reduced the incidence of exposure keratopathy among vulnerable critically ill patients, such as sedated, mechanically ventilated patients [20, 36, 37].

### Limitations of the study

#### Below are the common limitations that have been confronted in the current study

- ***Lack of a control group***: “The study did not include a randomized control group, which limits the ability to establish causality.”
- ***Short duration of the patient’s observation***: “The short follow-up period limited the ability to assess long-term outcomes.”
- ***Self-reported data***: “Part of the data was collected through self-report, which can be influenced by recall bias.”
- ***Limited generalizability***: “The study was conducted in a single hospital, which may not reflect broader population characteristics.”
- ***Lack of blinding***: “The Researcher was not blinded in the data collection of nurses’ attitudes and knowledge, which could introduce bias; however, the assessment of nurses’ eye care performance was conducted blindly, so there was no observer bias.”

## Conclusions

Attending eye care clinical guidelines training for intensive care nurses improved their knowledge, skills, attitudes, and reduced the incidence of eye complications among the critically ill patients admitted to the ICU. Thus, it is recommended that the ICUs’ administrative authorities in Egypt should adopt the eye care clinical guidelines and protocols for enhancing the quality of eye care and reducing eye problems.

### Recommendations

#### Implications for the profession and/or patient care

It was determined that intensive care nurses had limited knowledge and skills about eye care and that standard and guideline eye care practices were not performed. To increase the competence of intensive care nurses in eye care, this subject should be included in continuing in-service education and training, and integrating evidence-based eye care guidelines into practice. Educational initiatives and policy advancements will enhance nurses’ clinical competencies in eye care and promote patient safety.

### Implications for education

- The ECCG should be taught to undergraduate and postgraduate nursing students.

### Implications for research

- Replication of the study in different critical care settings on larger probability samples to help in the generalizability of findings.

## Data Availability

The datasets analyzed during the current study are available from the corresponding authors upon reasonable request.

## Abbreviations

EC: Eye Care
CCUs: Critical Care Units
ECCG: Eye Care Clinical Guidelines
OSDs: Ocular Surface Disorders
ECs: Eye Complications
SPRE: Surgeon Periorbital Rating of Edema
